# Biogeography of American Northwest Hot Spring A/B**′**-Lineage *Synechococcus* Populations

**DOI:** 10.3389/fmicb.2020.00077

**Published:** 2020-02-24

**Authors:** Eric D. Becraft, Jason M. Wood, Frederick M. Cohan, David M. Ward

**Affiliations:** ^1^Department of Land Resources and Environmental Sciences, Montana State University, Bozeman, MT, United States; ^2^Department of Biology, University of North Alabama, Florence, AL, United States; ^3^Biotechnology and Planetary Protection Group, Jet Propulsion Laboratory, California Institute of Technology, Pasadena, CA, United States; ^4^Department of Biology, Wesleyan University, Middletown, CT, United States

**Keywords:** ecotype, microbial species, population genetics, thermophilic *Synechococcus*, biogeography

## Abstract

Previous analyses have shown how diversity among unicellular cyanobacteria inhabiting island-like hot springs is structured relative to physical separation and physiochemical differences among springs, especially at local to regional scales. However, these studies have been limited by the low resolution provided by the molecular markers surveyed. We analyzed large datasets obtained by high-throughput sequencing of a segment of the photosynthesis gene *psaA* from samples collected in hot springs from geothermal basins in Yellowstone National Park, Montana, and Oregon, all known from previous studies to contain populations of A/B′-lineage *Synechococcus*. The fraction of identical sequences was greater among springs separated by <50 km than among springs separated by >50 km, and springs separated by >800 km shared sequence variants only rarely. Phylogenetic analyses provided evidence for endemic lineages that could be related to geographic isolation and/or geochemical differences on regional scales. Ecotype Simulation 2 was used to predict putative ecotypes (ecologically distinct populations), and their membership, and canonical correspondence analysis was used to examine the geographical and geochemical bases for variation in their distribution. Across the range of Oregon and Yellowstone, geographical separation explained the largest percentage of the differences in distribution of ecotypes (9.5% correlated to longitude; 9.4% to latitude), with geochemical differences explaining the largest percentage of the remaining differences in distribution (7.4–9.3% correlated to magnesium, sulfate, and sulfide). Among samples within the Greater Yellowstone Ecosystem, geochemical differences significantly explained the distribution of ecotypes (6.5–9.3% correlated to magnesium, boron, sulfate, silicon dioxide, chloride, and pH). Nevertheless, differences in the abundance and membership of ecotypes in Yellowstone springs with similar chemistry suggested that allopatry may be involved even at local scales. *Synechococcus* populations have diverged both by physical isolation and physiochemical differences, and populations on surprisingly local scales have been evolving independently.

## Introduction

Hot spring microbial mats have been used as model systems to demonstrate ecological diversification by sympatric adaptation to parameters that vary along well-established environmental gradients (Ward et al., 2012). For instance, a progression of unicellular cyanobacterial (*Synechococcus*) 16S rRNA genotypes (A″, A′, A, B′, and B, respectively) can be found along a thermal gradient in alkaline siliceous hot springs in Yellowstone National Park (YNP) (Ferris and Ward, 1997). This patterning led to the hypothesis that closely related *Synechococcus* populations might have different temperature adaptations and this was confirmed for isolates representative of these genotypes (Allewalt et al., 2006). Greater molecular resolution provided by a portion of the gene encoding a major photosystem I reaction center protein (*psaA* locus) demonstrated the existence of more closely related clades that were masked within 16S rRNA defined genotypes. These clades were shown to be ecologically distinct through their associations with different light environments at different depths in the upper 1 mm in these mats (Becraft et al., 2015). Isolates representative of *psaA* genotypes uniquely distributed along the vertical aspect of the mat were differently adapted to light intensity and quality (Nowack et al., 2015) and showed differences in gene content that may explain why these adaptations were observed (Olsen et al., 2015). In studies based on *psaA* variation, sequence clusters were demarcated into ecologically distinct populations (ecotypes) based on the Stable Ecotype model of species and speciation. Here, the Ecotype Simulation algorithm identified sympatric ecotypes satisfying the criteria for defining ecological species: they were ecologically distinct from one another and they were each ecologically homogeneous.

Because hot springs resemble islands, they also present an opportunity to understand the role of allopatric processes (i.e., physical isolation) in diversification of microbial populations (Papke et al., 2003). For many years, it was widely believed that barriers to the distribution of microbial species did not exist, and that “everything is everywhere, and the environment selects” (Baas Becking, 1934). In essence, microorganisms were thought to primarily evolve through sympatric means, and to be distributed globally without geographical barriers limiting their dispersal. Observations leading to such inferences were often based on a low-resolution taxonomy such as morphology (Finlay and Fenchel, 2004). However, morphology is often a poor indicator of species richness and can mask the genetic and ecological diversity that exists in nature. It can be challenging to study the spatial dynamics of microbial populations at large scale because (i) evolutionarily distinct organisms can share similar morphologies, (ii) cultivated organisms are not always representative of predominant natural populations (Ward et al., 1990), (iii) different taxa can have a variety of evolved dispersal mechanisms (McDougald et al., 2012), and (iv) the immense bacterial diversity that exists in nature can make identifying ecological species in their natural habitats difficult (Dykhuizen, 1998; Ward et al., 2006). Despite these complications, patterns of *Synechococcus* presence or absence in hot springs around the world have suggested a role for dispersal limitations in their biogeography (Castenholz, 1978, 1996).

Molecular technologies have allowed many researchers to infer a role for dispersal limitation in generating biogeographic patterns of microbes (Johnson et al., 2006; Martiny et al., 2006; Chase et al., 2018). Pathogenic and symbiotic microorganisms that are associated with specific eukaryotic hosts have been shown to be restricted to the geographic range of their hosts (Falush et al., 2003; Taylor et al., 2005; Peay et al., 2010). Geographic barriers have also been shown to isolate populations of hyperthemophilic Archaea (Whitaker et al., 2003; Whitaker, 2006). Likewise, thermophilic *Synechococcus* populations have been shown to be differentiated by geography (Ward and Castenholz, 2002; Papke et al., 2003; Ward et al., 2012). These studies provided evidence that geographic isolation has been an important factor in the diversification of microbial populations.

In the case of hot spring *Synechococcus* populations, Papke et al. (2003) showed that different *Synechococcus* 16S rRNA genotypes were predominant in Japanese, New Zealand and North American hot springs and were only rarely shared among springs in these locations. Analysis of the more rapidly evolving 16S-23S rRNA internal transcribed spacer region suggested regional variation in community members within Japanese and North American hot springs of different thermal basins, even in springs with very similar chemistry. Papke et al. (2003) characterized diversity using cloning and sequencing methods, so the sampling was limited. Furthermore, these genetic markers have been shown to be too slowly evolving to distinguish the most newly divergent ecological species (or ecotypes) of *Synechococcus* (Becraft et al., 2011, 2015; Melendrez et al., 2011). Additionally, these studies did not quantify how diversity within each hot spring was influenced by differences in the environmental parameters measured. These issues limit our ability to fully understand the relationship between local and regional communities, and thus the roles of dispersal and allopatric and sympatric processes in diversification of *Synechococcus* species.

In this study, we reanalyzed American Northwest samples collected by Papke et al. (2003) using high-throughput sequencing of amplicons of a segment of a more highly-resolving, protein-encoding locus (*psaA*) (Becraft et al., 2011, 2015). We have used Ecotype Simulation 2 (Wood et al., 2020) to identify the most newly divergent *psaA* segment sequence clusters that can coexist indefinitely because they are either ecologically distinct (ecotypes) or geographically isolated populations (geotypes), or both (Cohan and Perry, 2007). This permitted rigorous analysis of the distribution of A- and B′-like *Synechococcus* genetic diversity in hot springs that are separated by <1 to > 800 km, with the aim of gaining a better understanding of the role of allopatric processes in the diversification of *Synechococcus*.

## Materials and Methods

### Samples

**Figure 1:**
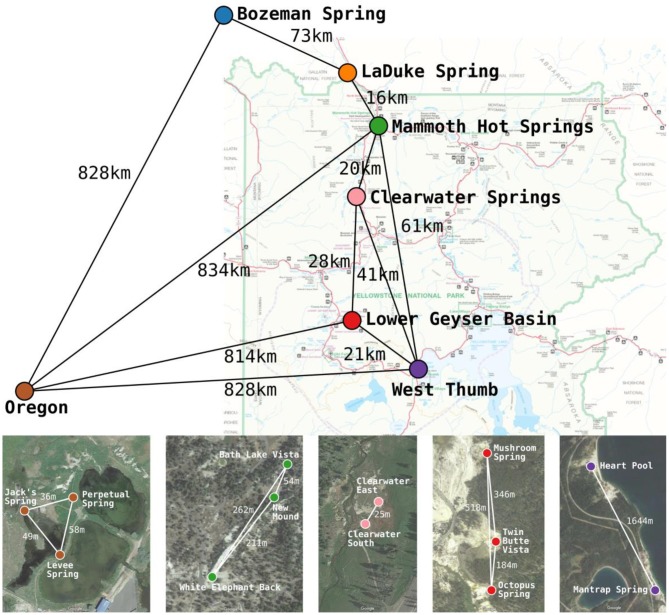
Approximate locations and geographic separation (km) among the 7 geographic regions studied across the Northwest United States by Papke et al. (2003). Satellite imagery insets show the approximate locations and geographic separation (m) among springs within Yellowstone National Park and Oregon basins. The satellite imagery was produced using Google Maps. Colors used for basins correspond between the map, satellite imagery, and later figures.

Duplicate mat samples and associated water samples for biogeographical analysis were collected from hot springs across the Northwest United States between 30 May and 8 July 1996 using a #4 cork borer (38.5 mm^2^) as reported by Papke et al. (2003) (summarized in Table 1 and Figure 1). Data from physical and chemical analyses for these springs are presented in Table 2 [see Papke et al. (2003) for analytical methods]. Samples were immediately frozen on dry ice in the field and kept frozen at −80°C until analysis in 2011. The sampling matrix included replicate springs within four geothermal basins in YNP, two Montana springs, one ~10 km north of (LaDuke) and the other ~150 km northwest of (Bozeman Hot Spring) YNP, and three hot springs in southwest Oregon. Samples used in biogeographical comparisons were collected at sites with near-neutral to alkaline pH in order to constrain ecological differentiation among populations, though some local ecological variation was present in the sample set. For instance, (i) samples were collected at different temperatures within Octopus Spring, YNP and Jack's Stream, Oregon, (ii) samples from Clearwater and Mammoth springs in YNP, and LaDuke Spring had lower pH levels than the other springs analyzed (5.2–6.9 compared to 8.1–8.8), and (iii) samples collected from Mammoth springs and LaDuke Spring had higher concentrations of calcium, magnesium, carbonate and sulfate compared to other Yellowstone springs.

**Table 1:** Samples analyzed in this study and the total number of genotypes and sequences analyzed.

**Spring**	**Barcode**	**Collection date**	**No. genotypes**	**No. sequences**
**LOWER GEYSER BASIN**				
Mushroom Spring A	64	31-May-96	774	2,063
Mushroom Spring B	82	31-May-96	756	1,696
Octopus Spring Low A	73	31-May-96	57	69
Octopus Spring Low B	74	31-May-96	830	2,018
Octopus Spring Med	65	31-May-96	793	1,906
Octopus Spring High	72	31-May-96	489	2,262
Twin Butte Vista A	77	31-May-96	711	1,954
Twin Butte Vista B	78	31-May-96	537	2,022
**CLEARWATER SPRINGS**				
Clearwater East A	91	30-May-96	391	3,208
Clearwater East B	92	30-May-96	389	4,136
Clearwater South	93	30-May-96	550	1,286
**WEST THUMB**				
Heart Pool A	75	31-May-96	549	1,678
Heart Pool B	76	31-May-96	590	1,866
Mantrap Spring A	85	31-May-96	637	1,562
Mantrap Spring B	86	31-May-96	58	86
**MAMMOTH HOT SPRINGS**				
Bath Lake Vista	62	30-May-96	280	4,904
New Mound A	89	30-May-96	228	4,526
New Mound B	90	30-May-96	278	4,703
White Elephant Back A	81	30-May-96	288	4,621
White Elephant Back B	84	30-May-96	317	4,710
LaDuke Spring	80	30-May-96	503	1,426
Bozeman Spring	83	8-Jul-96	319	3,932
**OREGON SPRINGS**				
Jack's Spring Low A	71	7-Jul-96	276	1,827
Jack's Spring Low B	87	7-Jul-96	260	1,782
Jack's Spring High	63	7-Jul-96	228	2,101
Levee Spring A	69	7-Jul-96	381	2,287
Levee Spring B	70	7-Jul-96	376	2,254
Perpetual Spring	67	7-Jul-96	383	2,014

**Figure 2:**
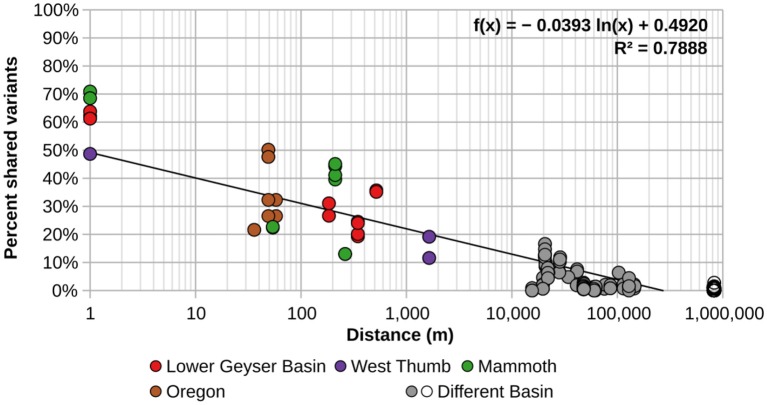
Percentage of shared A-like *Synechococcus psaA* gene segment HFS2 variants relative to separation between pairs of springs sampled. Each circle represents a pair of springs, with colored circles representing pairs from within the same basin. Gray circles represent pairs of springs from different basins within and around Yellowstone, while open circles represent comparisons between springs in Oregon with springs in and around Yellowstone National Park. The black trend line was calculated from all comparisons.

*Gray highlight emphasizes samples removed from analyses shown in Figure 2. Samples are separated by geothermal basin*.

**Table 2:** Physical and chemical parameters for hot springs sampled across the Northwestern United States.

**Spring**	**°C**	**pH**	**Latitude**	**Longitude**	**S^**2−**^**	**CaCO**	**Ca**	**Cl**	**Mg**	**SiO_**2**_**	**Na**	**SO_**4**_**	**K**	**As**	**B**	**Fe**	**Mn**	**Zn**
**LOWER GEYSER BASIN**																		
Mushroom Spring	58.9–60.7	8.3	44.54	−110.80	0	250	0.5	270	1	110	270	22	19	1.7	3	0.02	0.01	0.02
Octopus Spring Low	51.0–54.0	8.2	44.53	−110.80	0	300	0.5	310	1	120	300	21	15	1.5	3.5	0.02	0.01	0.02
Octopus Spring Med	54.0–64.0	8.2	44.53	−110.80	0.1	290	0.5	290	1	110	300	26	15	1.4	3.3	0.02	0.01	0.02
Octopus Spring High	64	8.5	44.53	−110.80	0.1	280	0.5	270	1	100	300	23	15	1.4	3.3	0.02	0.01	0.02
Twin Butte Vista	59.3–60.7	8.4	44.54	−110.80	0.6	270	0.5	320	1	110	290	21	13	2.1	3.3	0.02	0.01	0.02
**CLEARWATER SPRINGS**																		
Clearwater East	57.3–61.9	5.2	44.79	−110.74	1.3	8	6	130	1	40	74	26	16	0.68	2.7	0.53	0.04	0.02
Clearwater South	56.3–56.5	6.1	44.79	−110.74	0.2	31	7	140	1	45	94	33	20	0.67	2.8	0.14	0.1	0.02
**WEST THUMB**																		
Heart Pool	56.6–58.3	9.2	44.43	−110.58	20	480	0.5	350	1	120	410	44	23	2.1	4.3	0.02	0.01	0.02
Mantrap Spring	58.2–61.8	8.3	44.42	−110.57	0	350	0.5	180	1	94	270	29	12	1	2.5	0.02	0.01	0.02
**MAMMOTH HOT SPRINGS**																		
Bath Lake Vista	57.0–57.3	6.6	44.96	−110.71	39	720	330	170	63	27	120	600	55	0.94	4.7	0.02	0.01	0.02
New Mound	56.4	6.6	44.96	−110.71	25	720	350	160	65	27	120	600	54	0.82	4.3	0.07	0.01	0.02
White Elephant Back	59.7–60.0	6.9	44.96	−110.71	8.2	610	330	170	67	30	120	670	54	0.75	4.4	0.22	0.01	0.07
LaDuke Spring	59.8–60.2	6.7	45.10	−110.78	0	230	340	49	59	23	220	1,300	24	0.2	0.9	0.19	0.01	0.02
Bozeman Spring	57.2–57.5	8.8	45.66	−111.19	7.7	87	1	53	1	34	130	130	2	0.11	0.6	0.02	0.01	0.02
**OREGON SPRINGS**																		
Jack's Spring Low	49.7–50.2	8.4	42.22	−120.37	0.7	65	9	130	1	74	200	280	9	0.02	7.5	0.02	0.01	0.02
Jack's Spring High	59.8–61.2	8.3	42.22	−120.37	2.5	64	9	130	1	73	190	270	8	0.15	7.4	0.02	0.01	0.02
Levee Spring	59.5–61.8	8.1	42.22	−120.37	5.5	72	10	120	1	71	190	260	9	0.13	7.2	0.02	0.01	0.02
Perpetual Spring	60.1–60.9	8.2	42.22	−120.37	4.6	64	10	120	1	73	190	260	9	0.15	7.4	0.02	0.01	0.02

*Springs are separated by geothermal basin. Table modified from Papke et al. (2003). Units for chemical parameters are mg/L*.

### Molecular and Phylogenetic Analysis

DNA was extracted from mat samples, and a segment of the *psaA* locus was PCR-amplified using primers designed to target the *Synechococcus* A/B′-lineage. Amplicons were sequenced using Ti454-barcoding technology, as described in Becraft et al. (2015). Sequences were trimmed to 302 base pairs to obtain the maximum number of sequences, cleaned and analyzed to identify high-frequency sequences with ≥10 identical representatives across all combined samples (HFS10). This allowed us to restrict most analyses to sequences that were frequently detected. Also, analyses of HFS10s have proven valuable by increasing the sampling of variants within putative ecotypes, thereby enhancing our ability to demonstrate that sequence diversity within a putative ecotype is ecologically homogeneous (Becraft et al., 2015; Wood et al., 2020). However, we still used all sequences with >1 representative to analyze dispersal (see below).

Environmental *psaA* sequences similar to those found in publicly available A- and B′-like *Synechococcus* genomes [JA-3-3Ab, CP000239; and JA-2-3B′a(2-13), CP000240 respectively] (Bhaya et al., 2007) were split into separate A- and B′-like sequence datasets. BLASTn (Altschul et al., 1990) was used for comparison of these environmental sequences with the partial *psaA* HFSs found by Becraft et al. (2015); sequences with a top hit to an A-like HFS were assigned to the A-like dataset and those with a top hit to a B′-like HFS were assigned to the B′-like dataset. Separate A- and B′-like maximum-likelihood phylogenies were constructed from each sequence dataset with FastTree (Price et al., 2009). Some sample locations (from LaDuke Spring, Bozeman Spring, Bath Lake Vista, Clearwater Spring, Octopus Spring, Perpetual Spring, and Jack's Spring) could not be analyzed in duplicate due to failed sequencing reactions (see Table 1 for a list of samples). Sequences have been submitted to NCBI Genbank under accession numbers SAMN13631111–SAMN13631131.

### Putative Ecotype Demarcation

To analyze the distribution of HFS10 variation within ecological populations among chemically similar springs and regions, Ecotype Simulation 2 (ES2) was used to predict putative ecotypes (PEs) from the variation sampled. ES2 uses evolutionary simulation analysis to predict ecologically distinct or geographically isolated clusters in a phylogeny (Wood et al., 2020). Directions for download and instructions for the use of ES2 are freely available at https://github.com/sandain/ecosim. Because PEs demarcated by ES2 are not guaranteed to have the same HFS membership as previously described by Becraft et al. (2015) from a different dataset, they are named differently here. PEs demarcated in this study that contain members of PEs previously demarcated by Becraft et al. (2015) are indicated by enclosing the previously described PE name in parenthesis after the new PE name assigned by ES2 [e.g., PEB20 (B′12-1)].

### Abundance of HFSs Within Predominant PEs

The abundance of HFSs within predominant PEs (defined as those PEs making up >1% of sequences from at least one sample) was compared across environmental samples to measure (i) the reproducibility between biological replicates, (ii) the differences between communities in springs of different basins, (iii) similarity of communities within different springs within a basin, and (iv) the interchangeability of the various HFSs within a PE.

### Canonical Correspondence Analyses

The physical and chemical parameters measured at each spring (Table 2) were used as linear predictors of geographic and ecological differentiation among community members in canonical correspondence analyses (CCA) (Ter Braak, 1986; Legendre and Legendre, 1998) using software available from the R library vegan (Oksanen et al., 2013). The hfs-counter.pl script (available from https://github.com/sandain/pigeon) was used to count the abundance of HFS10 variants in each environmental sample to provide the data matrix analyzed by CCA. The plotting function used by Wood et al. (2020) was used here to display the distribution of HFS10 variants and predominant PEs in the CCA ordination space. This plotting function reports a *p*-value for each PE demarcation that represents the probability that the observed distribution of the PE in ordination space is in a tighter cluster than a randomly produced distribution of the same size in the same ordination space. The plotting function thus provides a test that a PE is ecologically distinct from the rest of the PEs in the sample and that the membership of the PE is ecologically interchangeable. PEs with only a single member cannot be tested in this way, so no *p*-value is provided.

To narrow the list of 18 physical and chemical parameters measured, a customized R script adapted from Roberts (2017) was written to find those parameters that significantly (*p* < 0.05) added to the CCA model. This custom script utilizes a forward step-wise approach, starting with the parameter that explained the most variation and stepping through other parameters until no further variation can be explained (stepCCA.R; available from https://github.com/sandain/R).

In order to visualize the variation of PEs along a single environmental gradient, a customized R script was written to run CCA and perform a weighted-density calculation for each PE in the ordination space. This script utilizes the R package vegan (Oksanen et al., 2013) along with the density function (ccadensity.R; available from https://github.com/sandain/R).

### Dispersal of *psaA* Variants Over Distance

Geographic distance was calculated from latitude and longitude of all pairwise sample combinations using software available at http://www.movable-type.co.uk/scripts/latlong.html. In this analysis, we used all sequences with ≥2 identical representatives across all combined samples (HFS2), as this would maximize the capacity to estimate geographic sharing of sequences that are extremely rare. The number of shared HFS2 variants across geographic distance was calculated by identifying all shared genotypes with 100% nucleotide identity present in each pairwise combination of samples of all springs across all basins. We removed from the analysis those sequences shaded in Table 1, as they were either too poorly sampled or were too extreme in temperature or pH, so that we could focus on the influence of physical separation more than ecological adaptation. The percentage of shared HFS2 variants for each sample-pair studied was determined by dividing two times the sum of the number of all HFS2 variants shared between samples by the total number of HFS2 variants in both samples.

$$\begin{array}{l} \frac{2*Shared_{SampleA\;\&B}}{Total_{SampleA}+Total_{SampleB}} \end{array}$$

Samples were arranged by geographic distance (m) from one another. Each data point represents the percentage of sequences in sample A shared with sample B and the geographic distance between the two samples, so each of the 21 samples has 20 separate pairwise comparisons for a total of 210 ($=\;\frac{21*20}{2}$) comparative data points. Distance between replicate samples from the same spring were not recorded, so a distance of 1 m was assumed to facilitate this comparison.

## Results

Samples collected by Papke et al. (2003) from various hot springs in the American Northwest known to contain A/B′-lineage *Synechococcus* (Figure 1) were sequenced, resulting in 68,899 *psaA* amplicon sequences (26,084 A-like and 42,455 B′-like; see Table 1 for sequence counts from individual springs). The B′-lineage was not detected in Oregon samples, likely due to sequence differences causing inefficient priming within a more evolved and thus variable genetic region. The A-lineage was poorly sampled in Mammoth springs, likely due to the lower temperatures of the springs sampled or inefficient priming (Table 1). We have organized the following presentation of results into sections that are intended to distill the essence of our observations in terms of the general patterning of diversity relative to geographical separation of springs, phylogenetic relatedness of variants from different locations, and ecological parameters.

### Pairwise Sharing of Sequences Across Springs

The degree to which sequence HFS10 variants in samples were found among other samples is reported in Table 3 (below diagonal). In this section we consider the sharing of sequences across springs, from local to increasingly distant scales. Replicate samples showed between 66.3 and 95.7% sharing (average 74%), with the exception of two pairs of samples in which one replicate was poorly sampled (Table 1). Among replicate samples there was a clear relationship between the number of sequences sampled and percent shared sequences (Supplemental Figure 1), so that the degree of sharing is likely to have been underestimated.

**Table 3:** Pairwise comparison of percentage of identical shared HFS10 sequences (below diagonal) and number of shared HFS2 sequences in the paired sample with the fewest sequences (above diagonal) for springs sampled in this study.

**Spring**	**Mushroom Spring A**	**Mushroom Spring B**	**Octopus Spring High**	**Octopus Spring Low A**	**Octopus Spring Low B**	**Octopus Spring Low Medium**	**Twin Butte Vista A**	**Twin Butte Vista B**	**Clearwater East A**	**Clearwater East B**	**Clearwater South**	**Heart Pool A**	**Heart Pool B**	**Mantrap Spring A**	**Mantrap Spring B**	**Bath Lake Vista Annex**	**New Mound Spring A**	**New Mound Spring B**	**White Elephant Back A**	**White Elephant Back B**	**La Duke**	**Bozeman Spring**	**Jack's Spring High**	**Jack's Spring Low A**	**Jack's Spring Low B**	**Levee Spring A**	**Levee Spring A**	**Perpetual Spring**
Mushroom Spring A	–	921	25	26	333	364	262	134	56	35	39	65	63	40	9	1	3	1	1	2	3	6	3	0	0	2	0	0
Mushroom Spring B	83.0	–	22	24	338	355	266	122	47	24	28	43	42	28	9	5	7	6	4	5	3	8	3	0	0	2	0	0
Octopus Spring High	10.3	10.3	–	0	3	27	10	21	116	1	1	1	3	40	2	0	1	1	0	0	0	0	1	0	0	0	0	0
Octopus Spring Low A	23.8	23.8	0.0	–	47	29	20	12	2	2	3	4	5	6	2	1	1	0	0	0	2	3	0	0	0	1	0	0
Octopus Spring Low B	60.7	63.7	4.4	39.4	–	555	197	101	29	29	59	44	38	51	25	17	8	8	5	4	23	63	2	0	0	3	0	0
Octopus Spring Medium	62.8	64.7	11.2	25.6	64.4	–	338	239	149	129	84	138	127	56	8	6	5	4	2	3	16	24	3	0	0	3	0	0
Twin Butte Vista A	54.1	50.8	9.9	18.0	46.2	61.5	–	918	472	451	55	478	366	41	9	2	2	1	1	2	5	5	3	0	0	2	0	0
Twin Butte Vista B	45.1	43.9	12.8	12.1	35.6	51.1	83.5	–	939	916	48	581	358	47	10	2	2	1	1	2	2	0	3	0	0	2	0	0
Clearwater East A	18.6	21.1	22.7	4.6	16.0	28.6	34.2	34.3	–	2,707	252	567	348	36	3	113	30	31	26	30	5	8	3	0	0	1	0	0
Clearwater East B	12.5	15.3	2.5	5.7	16.6	23.8	26.4	27.4	67.7	–	245	558	342	26	3	134	19	21	18	19	7	14	3	0	0	1	0	0
Clearwater South	19.3	17.9	2.5	8.5	24.7	27.6	23.1	25.4	49.1	53.9	–	40	37	25	4	13	3	5	1	1	44	78	3	0	0	0	0	0
Heart Pool A	25.3	22.7	2.3	10.5	23.8	26.8	29.6	27.6	28.8	23.4	17	–	743	55	5	0	2	1	0	1	2	5	5	2	2	2	2	2
Heart Pool B	23.0	23.0	4.8	13.5	17.4	25.8	28.6	28.1	25.7	17.4	19.4	63.3	–	85	5	0	2	1	0	0	1	4	3	0	0	0	0	0
Mantrap Spring A	14.0	14.0	17.7	8.7	12.5	13.3	12.5	13.8	11.5	4.6	6.8	23.7	44.0	–	41	0	1	1	0	0	3	2	2	0	0	1	0	0
Mantrap Spring B	5.4	7.1	8.3	5.3	7.1	6.7	6.2	7.1	2.7	3.6	7.0	6.5	10.0	40.0	–	0	1	1	0	0	0	0	2	0	0	0	0	0
Bath Lake Vista Annex	1.6	4.7	0.0	3.8	10.9	6.0	1.8	2.0	22.7	22.5	13.9	0.0	0.0	0.0	0.0	–	442	446	365	366	1	6	0	0	0	1	0	0
New Mound Spring A	4.4	7.4	2.8	3.3	8.8	7.0	3.3	3.7	18.8	10.1	7.5	4.7	4.8	2.6	4.3	51.6	–	4,324	2,909	2,861	0	7	1	0	0	1	0	0
New Mound Spring B	1.5	4.5	2.9	0.0	5.9	4.3	1.7	1.9	16.8	10.3	7.6	2.4	2.4	2.6	4.3	52.5	95.7	–	2,942	2,907	0	6	1	0	0	1	0	0
White Elephant Back A	1.5	4.6	0.0	0.0	4.5	1.4	1.7	1.9	8.7	8.0	2.6	0.0	0.0	0.0	0.0	34.5	69.7	73.8	–	4,328	0	6	0	0	0	1	0	0
White Elephant Back B	3.0	4.5	0.0	0.0	3.0	4.3	3.4	3.8	17.0	10.4	2.6	2.4	0.0	0.0	0.0	30.0	70.6	74.6	87.5	–	0	6	0	0	0	1	0	0
La Duke	4.9	3.3	0.0	8.3	8.1	4.7	3.7	4.2	7.2	9.1	1.9	2.8	2.9	3.1	0.0	4.1	0.0	0.0	0.0	0.0	–	30	0	0	0	1	0	0
Bozeman Spring	3.4	5.1	0.0	9.1	13.4	9.6	3.9	0.0	7.6	12.9	15.9	2.9	3.0	6.6	0.0	4.4	3.8	3.8	4.1	3.9	5.0	–	0	0	0	0	0	0
Jack's Spring High	2.9	2.9	2.7	0.0	1.4	2.8	3.3	3.6	4.1	4.9	4.9	9.2	4.7	2.5	4.1	0.0	2.8	2.8	0.0	0.0	0.0	0.0	–	787	754	1,507	1,302	664
Jack's Spring Low A	0.0	0.0	0.0	0.0	0.0	0.0	0.0	0.0	0.0	0.0	0.0	3.9	0.0	0.0	0.0	0.0	0.0	0.0	0.0	0.0	0.0	0.0	49.4	–	1,412	850	872	1,331
Jack's Spring Low B	0.0	0.0	0.0	0.0	0.0	0.0	0.0	0.0	0.0	0.0	0.0	3.8	0.0	0.0	0.0	0.0	0.0	0.0	0.0	0.0	0.0	0.0	54.3	91.6	–	790	771	1,331
Levee Spring A	2.6	2.6	0.0	2.5	3.9	3.7	2.9	3.1	1.7	2.0	0.0	3.8	0.0	2.1	0.0	2.5	2.2	2.3	2.4	2.3	2.6	0.0	70.3	52.8	56.9	–	1,641	798
Levee Spring B	0.0	0.0	0.0	0.0	0.0	0.0	0.0	0.0	0.0	0.0	0.0	4.0	0.0	0.0	0.0	0.0	0.0	0.0	0.0	0.0	0.0	0.0	75.0	56.3	60.4	91.4	–	825
Perpetual Spring	0.0	0.0	0.0	0.0	0.0	0.0	0.0	0.0	0.0	0.0	0.0	4.0	0.0	0.0	0.0	0.0	0.0	0.0	0.0	0.0	0.0	0.0	50.0	84.4	90.6	57.1	58.8	–

*Gray highlight emphasizes possible evidence of sharing between springs in Oregon and the Greater Yellowstone Ecosystem*.

Comparisons at the local scale yielded differences that were likely due to the effects of environmental parameters. This was suggested by noting that samples from different temperature sites showed less sharing than replicate samples from the same temperature. For instance, within Octopus Spring, high-temperature samples shared only 11.2% of sequences with the medium-temperature sample and 0–4.4% (average 2.2%) of sequences with low temperature samples; medium- and low-temperature samples shared 25.6–64.4% (average 51.8%) of sequences. Within Jack's Spring, a high-temperature sample shared 49.4–54% (average 45.5%) of sequences with low-temperature samples, substantially lower than the 91.6% of shared sequences in low-temperature replicate samples.

Geographic distance was important in predicting the sharing of sequences. Among springs of roughly similar temperature and pH of 6.1–9.2 (unhighlighted in Table 1), sharing of sequences between springs of the same basin was lower than between replicate samples from the same spring. For example, sharing ranged from 43.9 to 64% between springs of the Lower Geyser Basin, 23–44% in West Thumb springs, 30–74.6% in Mammoth springs, and 52.8–87.4% in Oregon springs. Samples from springs in different basins shared a yet lower percentage of sequences, with 0–29.6% among springs in different Yellowstone basins. Sharing between Yellowstone and Oregon springs was even lower, ranging from 0 to 3.7%.

In order to better visualize the general effect of physical separation, the percentages of shared sequences were compared to the distances separating them (Figure 2). Additionally, these analyses were performed using the minimum number of sequences needed to observe sharing of sequences in more than one spring (HFS2). There was obvious spatial restriction on the distribution of A/B′-lineage *Synechococcus* among the springs we studied.

### Phylogenetic Relatedness of HFS10 Sequences From Springs of Different Locations

**Figure 3:**
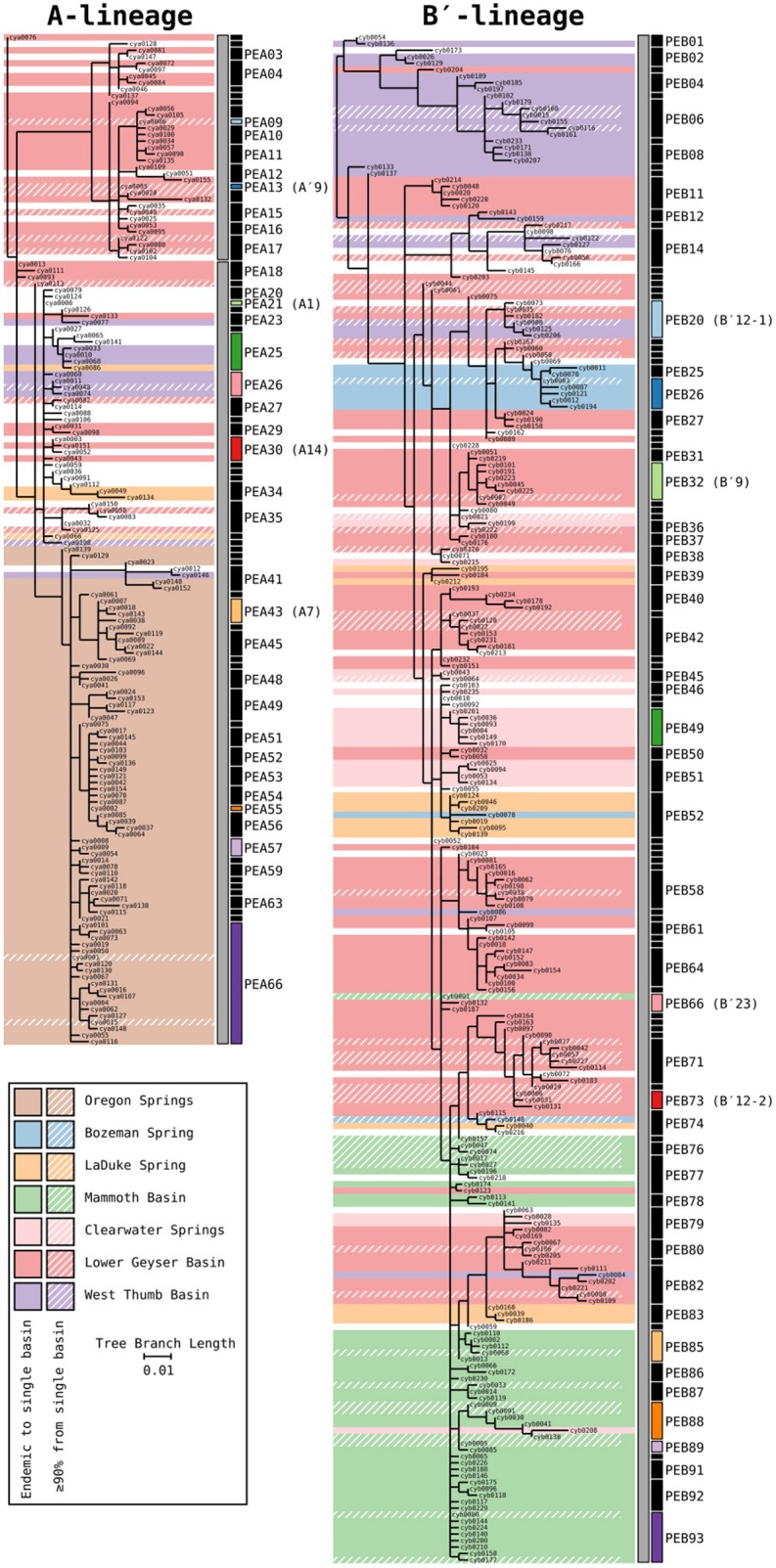
Phylogenies of 16S rRNA defined A′ (PEA1-PEA17), A (PEA18-PEA66), and B′-like *Synechococcus* (gray bars) based on partial *psaA* gene HFS10 variants from Yellowstone National Park, Montana, and Oregon Springs, with putative ecotype (PE) demarcations based on Ecotype Simulation 2 (ES2). Shaded regions of the trees mark branches that are endemic to a single basin. Regions with a diagonal hatch mark branches predominantly found in a single basin (>90%). The ES2 PE demarcations are displayed next to each tree as vertical black and colored bars, with colored bars representing predominant PEs analyzed in detail. Demarcation colors are reused between the A- and B′-like phylogenies and are the same in later figures. Labels are skipped for most PEs with only a single member.

Phylogenetic trees for the *Synechococcus* A- and B′-lineages are shown in Figure 3. Our analyses yielded 66 A-like and 93 B′-like PEs. A-like sequences from Oregon (highlighted brown in Figure 3) were quite divergent from Yellowstone and Montana sequences. Within Yellowstone and within Montana, large segments of the trees colored differently indicate significant phylogenetic divergence among different basins. For instance, B′-like sequences highlighted in green represent those from the Mammoth Hot Springs basin, sequences highlighted in purple represent those from the West Thumb basin, and sequences highlighted in blue and light orange represent those from Bozeman Hot Springs and LaDuke hot spring, respectively. The shading in the tree compared to the PE demarcation bars to the right of each tree demonstrated that a large number of detected PEs were endemic or nearly endemic (≥90%) to a single basin (45 of 66 A-like PEs and 62 of 93 B′-like PEs).

### Abundances of HFS10 Sequences Within PEs

**Figure 4:**
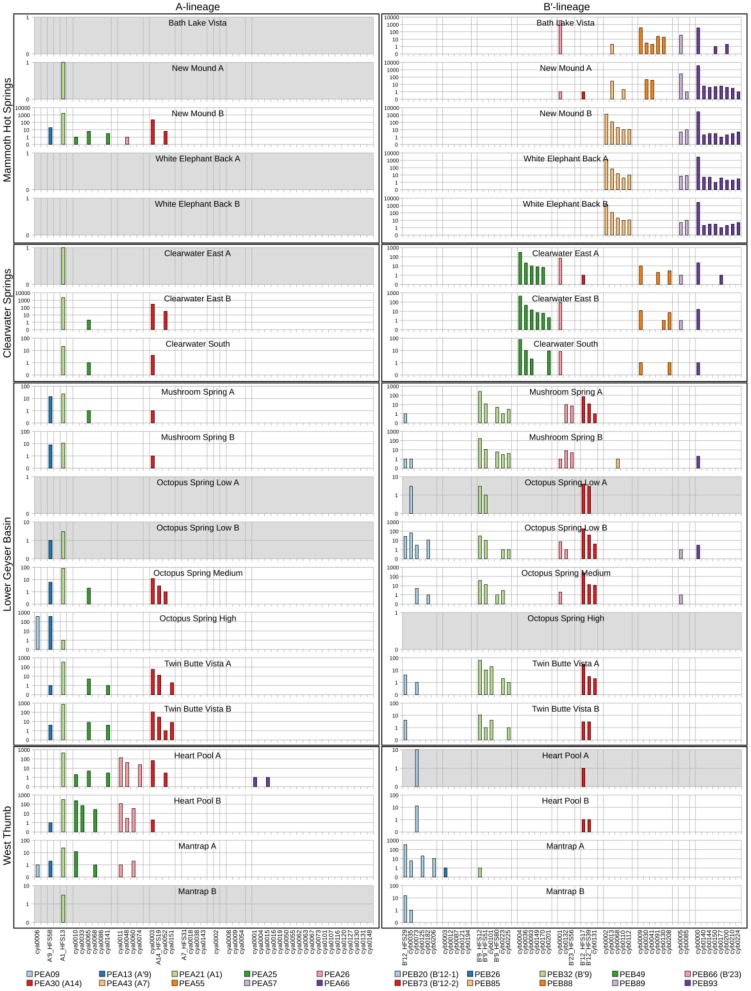
Abundance of HFS10 *psaA* sequence segments in predominant *Synechococcus* A- and B′-like putative ecotypes (PEs) for springs within Yellowstone National Park. Poorly sampled springs are shaded gray.

**Figure 5:**
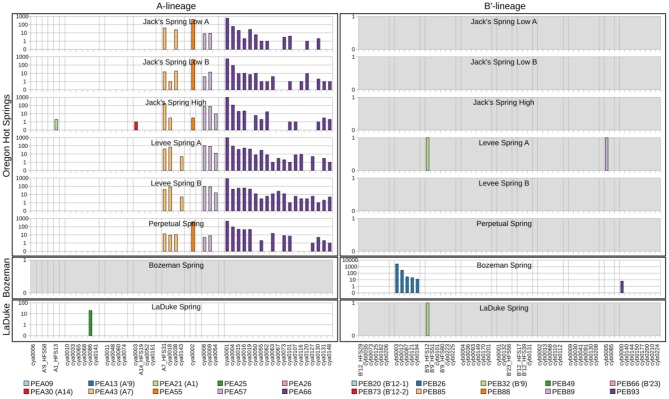
Abundance of HFS10 *psaA* sequence segments in predominant *Synechococcus* A- and B′-like putative ecotypes (PEs) for springs outside of Yellowstone National Park. Poorly sampled springs are shaded gray.

Because each unique sequence was used only once in the phylogeny, it was only when the frequencies of HFS10 variants within predominant PEs were taken into account that the degree of endemism among different basins and springs could be fully appreciated. Figures 4 and 5 present the number of occurrences (log scale) of each HFS10 variant within predominant PEs detected in each mat sample. With this presentation, it was possible to examine population and community structure in terms of the similarity across springs within a basin and the differences among springs in different basins (compare A and B′ lineages among basins in YNP in Figure 4 with those in Montana and Oregon in Figure 5).

Comparison of paired samples, especially those with deeper sequence coverage (Mushroom Spring, Twin Butte Vista, Jack Stream and Levee Spring in the A-lineage; White Elephant Back, Clearwater East, and Twin Butte Vista in the B′-lineage) demonstrated the fidelity of the approach to reproducibly sample the populations present. Variants classified to a given PE were often found in all springs within a basin. Ocassionally the HFS10 variants within a PE varied between replicate samples, including New Mound Spring [differences in variants of PEB85 and PEB88] and Mushroom Spring [differences in PEB73 (B′12-2)]. In most cases, variability in sampling and the low abundances of some HFS10 variants prevented us from making statistically significant inferences about population genetics within PEs.

### Regional Endemism Among PEs

**Figure 6:**
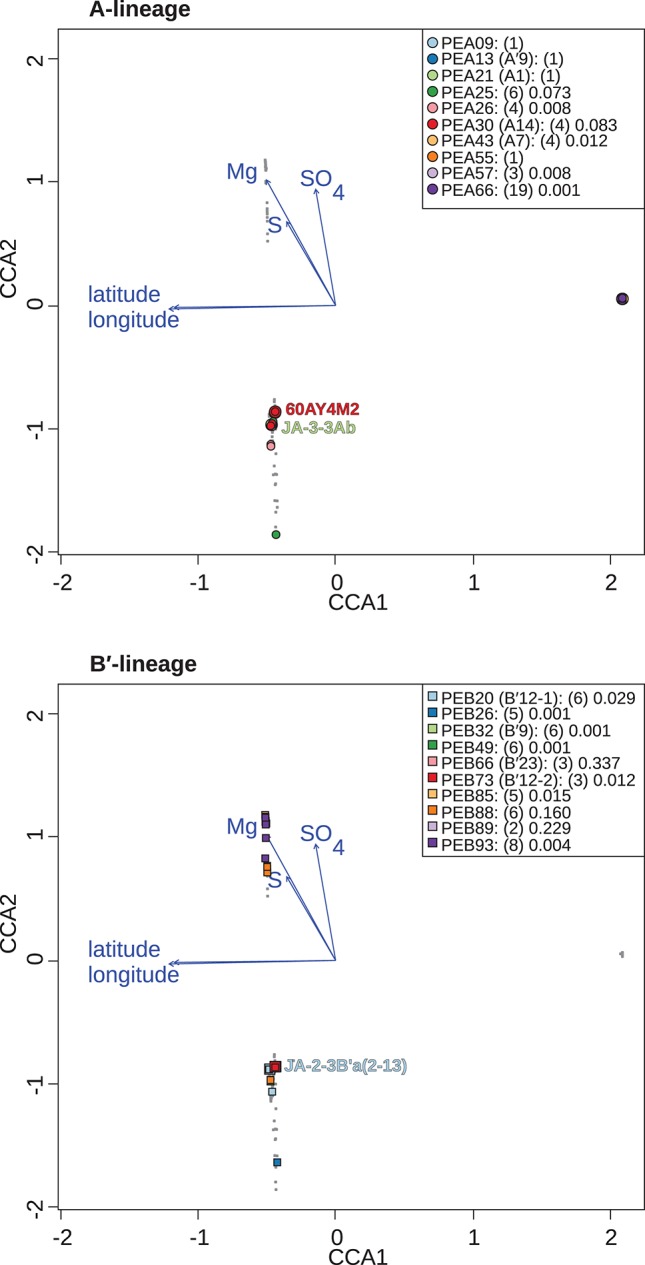
Canonical correspondence analyses of *Synechococcus* A- **(A)** and B′-like **(B)** *psaA* sequence segment HFS10 diversity recovered from Yellowstone National Park, Montana, and Oregon hot springs. Larger symbols represent sequences described previously by Becraft et al. (2015). *Synechococcus* strains JA-3-3Ab, 60AY4M2, and JA-2-3B'a(2-13) share *psaA* sequence segments with high-frequency sequences (HFSs) in putative ecotypes (PEs) PEA21 (A1), PEA30 (A14), and PEB20 (B′12-1), respectively, and are labeled on each plot. **Small gray dots** represent HFSs from lower-abundance PEs or from the other lineage. Directional arrows represent the vector of influence of each of the significant parameters on the ordination space. PE names in the legend are followed by the number of HFSs making up the PE in parenthesis and a p-value that represents the probability that the observed PE cluster is randomly produced.

There were several examples of predominant PEs that were endemic to different regions. For instance, PEB49 was found only in Clearwater Springs (YNP), and Oregon PEA55 and PEA57 were endemic to Oregon springs (Figure 6). PEA66 is endemic with respect to the springs in Oregon, with the exception that it was also detected in one spring in YNP. As shown by the purple bars in Figure 4 (A-lineage, bottom), a single sequence of each of these variants was detected in one of the Heart Pool samples, whereas hundreds of sequences of PEA66 HFS variants were detected in all Oregon springs. There were several other examples in the dataset, in which variants that were abundant in YNP springs were detected rarely (i.e., 1–2 sequences) in Oregon Springs. For instance, rare examples of PEA21 (A1) and PEA30 (A14) variants were detected in a Jack Spring high sample, and single sequences of PEB32 (B′9) and PEB89 were recovered from a Levee Spring sample.

### Near Endemism Among PEs of Greater Yellowstone Ecosystem Basins

Similarly, several PEs were nearly endemic to a single basin within the Greater Yellowstone Ecosystem. For instance, (i) PEA26 appeared endemic to West Thumb Basin, except for a single instance of a variant detected in New Mound Spring within the Mammoth Hot Springs basin, (ii) PEB26 appeared endemic to Bozeman Hot Spring, except for a single instance of a variant detected in Mantrap Spring from West Thumb Basin, (iii) PEB32 (B′9) appeared endemic to Lower Geyser Basin springs, except for a single instance of a variant detected in a West Thumb spring, (iv) PEB73 (B′12-2) also appeared endemic to Lower Geyser Basin springs, except for two variants detected at very low quantity in Heart Pool, Clearwater, and New Mound springs, (v) PEB85 appeared endemic to Mammoth springs, except for a single instance of a variant detected in Mushroom Spring, and (vi) PEB89 appeared endemic to Mammoth springs, except for the detection of a single HFS variant in Octopus Spring and Clearwater Spring samples. The near endemism of PEA26, PEB32 (B′9), and PEB73 (B′12-2) were especially noteworthy because all other PEs abundant in the Lower Geyser Basin springs were also abundant in the West Thumb springs.

### Within-Basin PE Endemism

In general, PEs contained the same HFS10 variants in different springs within the same basin. Exceptions included the absence of (i) PEB66 (B′23) and PEB88 in White Elephant Back Spring in Mammoth Basin, and (ii) PEB66 (B′23) from Twin Butte Vista Spring in the Lower Geyser Basin, but these examples suffer from the lack of reproducibility in New Mound Spring paired samples and the relatively poor degree of amplification in Twin Butte Vista samples. Interestingly, the most abundant HFS10 variants in PEB20 (B′12-1) were different in Heart Pool and Mantrap Spring. Although three of the four samples were low in coverage, the result was reproducible in replicate samples.

### PEs Reliably Detected in More Than One Greater Yellowstone Ecosystem Basin

Some PEs demonstrated a more cosmopolitan distribution and were found at a frequency of >10 per sample in samples from different basins. Notably, A-lineage PEA21 (A1), PEA25, and PEA30 (A14) were found in all Yellowstone basins sampled, and A-lineage PEA13 (A′9) was found in all but the samples from Clearwater springs. Similarly, B′-lineage PEB20 (B′12-1) could be found in Lower Geyser Basin and West Thumb samples, and B′-lineage PEB88 and PEB93 could be found in samples from Mammoth Hot Springs and Clearwater Springs.

### Possible Evidence of Long-Distance Dispersal

In a few cases, there was evidence of possible historical (i.e., past) dispersal. For instance, one relatively low-abundance A-like PE, PEA41, was embedded in a clade that was mainly endemic to Oregon (see A-like PEA38-PEA66 in Figure 3). This PE contained two HFS10 variants that were found only in the Oregon springs and two HFS10 variants that were found only in Yellowstone springs. The evidence is weakened when resolution was enhanced by examining HFS2 variants. Only 2–3 HFS2 variants were observed as being shared between YNP and Oregon samples (Table 3, above diagonal). Only four samples (Heart Pool A and B, Jack's Spring high, and Levee Spring A) show such low-level sharing and, in cases where samples are replicated, replicates do not always show sharing. This suggests that these “shared” sequences may be artifacts of contamination between wells on sequencing plates. Likewise, PEB52 shows evidence of a potential dispersal event from LaDuke Spring to Bozeman Hot Spring. Note that a HFS10 variant which was only found in Bozeman Hot Spring, was embedded within a clade of LaDuke Spring sequence variants (Figure 3). In total, 30 HFS2 variants (see Table 3, above diagonal) were shared between LaDuke Spring and Bozeman Hot Spring, increasing the likelihood that there was an exchange of variants between these springs.

### Correlation of HFS10 Sequence Variants in PEs With Geographic Separation and Physical/Chemical Parameters

Canonical correspondence analyses were run on the data matrix using 3 physical and 15 chemical parameters (Table 2) as potential linear predictors of ecological differentiation among populations sampled. Five physical and chemical parameters added significantly (*p* < 0.05) to the CCA model: longitude, latitude, magnesium, sulfate, and sulfide (Table 4 and Figure 6).

**Table 4:** Analyses of constrained variability and significance of parameters (*p* < 0.05) in the canonical correspondence analyses model.

**Parameter**	**Constrained**		**Significant w/o**
	**variability (%)**	**Significant**	**Oregon samples**
Longitude	9.46	Yes	No
Latitude	9.42	Yes	No
Mg	9.26	Yes	Yes
K	9.26	No	No
Ca	9.24	No	No
B	9.23	No	Yes
SO_4_	9.10	Yes	Yes
CaCO	8.61	No	No
SiO_2_	7.84	No	Yes
As	7.62	No	No
S^2−^	7.37	Yes	No
CI	7.03	No	Yes
pH	6.51	No	Yes
Na	6.34	No	No
Mn	6.23	No	No
Fe	5.66	No	No
Zn	5.13	No	No
Temperature	3.95	No	No

When all samples across the American Northwest were analyzed together, longitude and latitude were among the most important parameters (Table 4). Given the distance between Oregon and the rest of the environmental samples (>800 km; see Figure 1), it was not surprising that longitude and latitude correlated with separation of PEs endemic to Oregon. A-like PEA43 (A7), PEA57, and PEA66 were in very tight clusters with *p* < 0.05 stacked on top of each other on the right side of Figure 6A. Oregon PEs were well separated from those in and around Yellowstone [see A-like PEA26 or B′-like PEB20 (B′12-1), PEB26, PEB32 (B′9), PEB49, PEB73 (B′12-2), PEB85, and PEB93] with *p* < 0.05 that were spread out along the CCA2 axis in Figure 6 in the ordination space. Magnesium, sulfide, and sulfate concentrations separated the PEs in and around Yellowstone. The majority of PEs with more than one HFS member showed restricted distributions in the ordination space [e.g., A-like PEA25 and PEA30 (A14) with *p* < 0.1, and PEA26, PEA43, PEA57, and PEA66 with *p* < 0.05; and B′-like PEB20 (B′12-1), PEB26, PEB32 (B′9), PEB49, PEB73 (B′12-2), PEB85, and PEB93 with *p* < 0.05 in Figure 6].

**Figure 7:**
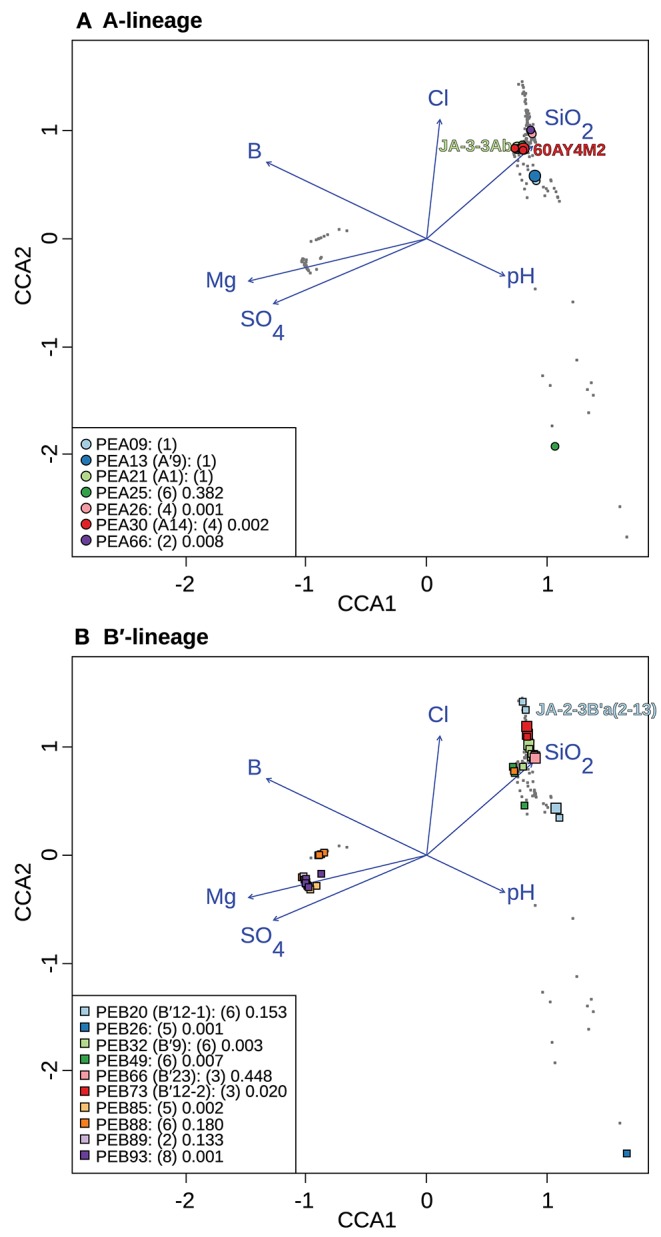
Canonical correspondence analyses of *Synechococcus* A- **(A)** and B′-like **(B)** *psaA* sequence segment HFS10 diversity recovered from Yellowstone National Park and Montana hot springs. Larger symbols represent sequences described previously by Becraft et al. (2015). *Synechococcus* strains JA-3-3Ab, 60AY4M2, and JA-2-3B'a(2-13) share *psaA* sequence segments with high-frequency sequences (HFSs) in putative ecotypes (PEs) PEA21 (A1), PEA30 (A14), and PEB20 (B′12-1), respectively, and are labeled on each plot. **Small gray dots** represent HFSs from lower-abundance PEs or from the other lineage. Directional arrows represent the vector of influence of each of the significant parameters on the ordination space. PE names in the legend are followed by the number of unique HFSs making up the PE in parenthesis and a p-value that represents the probability that the observed PE cluster is randomly produced.

To test whether longitude and latitude remained as significant determinants of biogeographical distribution even within a region, the community data matrix was also analyzed without the Oregon samples. In this case, latitude and longitude were not significant to the CCA model. This resulted in magnesium, boron, sulfate, silicon dioxide, chloride, and pH adding significantly to the CCA model (Table 4 and Figure 7). Chloride, boron, and pH were associated with the separation of PEs that were shared among the Lower Geyser Basin, Clearwater, and West Thumb basins [e.g., A-like PEA26, PEA30 (A14), and PEA66 *p* < 0.01; and B′-like PEB32 (B′9), PEB49, and PEB73 (B′12-2) *p* < 0.05] from those found in Bozeman and LaDuke hot springs (e.g., B′-like PEB26 *p* < 0.01; Figure 7). Magnesium, sulfate, chloride, and silicon dioxide separated the predominant B′-like PEs endemic to Mammoth hot springs (e.g., PEB85, PEB88, PEB89, and PEB93) from the Lower Geyser Basin and West Thumb hot springs (Figure 7).

**Figure 8:**
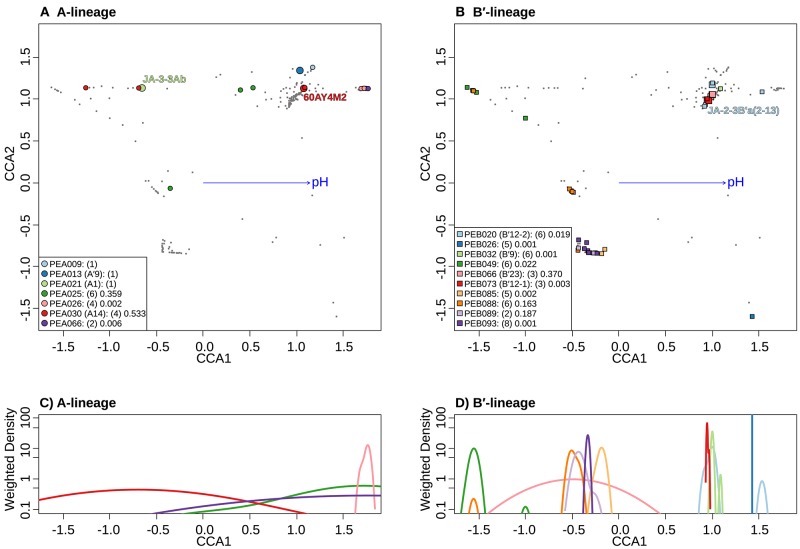
Canonical correspondence analyses (CCA) relative to pH of *Synechococcus* A- **(A)** and B′-like **(B)** HFS10 *psaA* sequence segments within predominant putative ecotypes (PEs) recovered from Yellowstone National Park and Montana hot springs. Weighted density of predominant A- **(C)** and B′-like **(D)** PEs in the ordination space defined by the pH vector. Larger symbols in A and B represent sequences described previously by Becraft et al. (2015). *Synechococcus* strains JA-3-3Ab, 60AY4M2, and JA-2-3B'a(2-13) share *psaA* sequence segments with high-frequency sequences (HFSs) in putative ecotypes (PEs) PEA21 (A1), PEA30 (A14), and PEB20 (B′12-1), respectively, and are labeled in A and B. Small gray dots in **(A,B)** represent HFSs from lower-abundance PEs or from the other lineage. Directional arrows represent the vector of influence of pH on the ordination space in **(A,B)**. PE names in the legend are followed by the number of unique HFSs making up the PE in parenthesis and a p-value that represents the probability that the observed PE cluster is randomly produced.

Separate CCA analyses of pH provided evidence suggesting that PEs endemic to the most alkaline and acidic springs sampled may be adapted to high and low pH. PEA26 and PEB26, which are endemic to Heart Pool (pH 9.2) and Bozeman Spring (pH 8.8), respectively, form significantly tighter clusters at the high end of the pH range in the ordination space (Figures 8A,B). Similarly, PEB49, which is endemic to Clearwater Springs (pH 5.2–6.1), forms a significantly tight cluster at the lower end of the pH range. By analyzing the weighted density of these PEs, this pH relationship becomes more apparent (Figures 8C,D). PEA26 (pink) and PEB26 (blue) formed distinct density curves on the alkaline end of the pH range, while PEB49 (green) formed a distinct density curve on the acidic end of the pH range. Interestingly, PEB88, PEB89, PEB93, and PEB85 formed distinct density curves with minimal overlap and different pH optima near the center of the pH range analyzed. PEB20 was separate from other PEs in Heart Pool and Lower Geyser Basin. One sequence variant of this PE (cyb0073) was most abundant in Heart Pool and not present Mantrap Spring, though it was identified in low abundance in Octopus Spring and Twin Butte Vista Spring of the Lower Geyser Basin. Sequence cyb0073 is a clear outlier when examining the density curve related to pH (far right blue square in Figure 8B), indicating a possible lumping of two ecotypes with distinct pH adaptations.

## Discussion

Allopatric and sympatric processes both appear to have played a role in the diversification of A/B′-lineage *Synechococcus* inhabiting the hot springs of the American Northwest. The Yellowstone National Park hot spring “archipelago” has different communities from geographically distinct hot springs in Oregon. The distances between springs serve as a physical barrier that limits dispersal. Many of the predominant PEs found in this study are endemic to a single geothermal basin, with geographical separation explaining the highest degree of differences in populations among basins separated by >50 km.

CCA indicates that magnesium, sulfate, and sulfide serve as good linear predictors of the differences in community composition among all samples. Magnesium, sulfate, boron, pH, chloride, and silicon dioxide serve as good linear predictors when analyzing just the Yellowstone and Montana samples. Although these parameters may not themselves drive diversification of *Synechococcus* PEs, they may co-vary with parameters that do. For instance, temperature was not identified as a significant parameter in these analyses, though variation along the chloride vector may correlate with temperature, as evaporation along the thermal gradient of the effluent channel causes chloride concentration to rise (Nordstrom et al., 2005). This can be noted in the distribution relative to the chloride vector of A- and B′-like PEs (Figure 7). CCA placed B′-like PEs, which are found in cooler water, further along the chloride vector, correlating with the lower temperature. By comparison, CCA placed A-like PEs, which are found in warmer water, at a lower position along the chloride vector, correlating with higher temperature.

Becraft et al. (2015) previously demonstrated differentiation among PEs based on resource availability and physical conditions within the mat community of Mushroom Spring, where PEs were separated spatially along the vertical aspect of the mat. Light is quickly attenuated in the microbial mat environment, altering the intensity, and quality of light available for photosynthesis for subsurface PEs. The concentration of dissolved minerals, and other ions or chemicals that are important inputs or outputs of *Synechococcus* metabolism also changes with depth over a diel cycle (Revsbech et al., 2016). In the present study we were unable to study depth as a parameter associated with distribution, since analyses were performed on bulk mat samples that had been collected before PEs adapted to different light environments had been discovered. However, it seems likely that these environmental parameters have similar influences on genetic diversification of populations in other springs and environments (Johnson et al., 2006). Though CCA evidence is correlative, the significance of pH in CCA analysis, combined with the endemicity of PEs in the most acid and alkaline springs studied, leads to the hypothesis that pH may be another parameter that drives sympatric speciation in these populations.

The differences in PEs and PE abundances could be due to the effects of island biogeography, where *Synechococcus* ecotypes migrate across relatively large distances, and then adapt allopatrically to their separate environments (MacArthur and Wilson, 2001). This would first involve migration between springs within a basin, and less frequently migrations between basins, allowing for the colonization of new hot springs. Then, once in a separate locations, the populations could accumulate neutral genetic changes and ultimately adaptive genetic changes in response to the different chemistries and communities of the new spring. Differences in variants within a PE among nearby springs may stem from differences in dispersal among closely related populations in the springs, where the original individual that migrated to a new spring initiated a founder effect, causing a bottleneck in genetic diversity that subsequently arose from that founder variant. We may have observed an example of this by noting different dominant variants in PEB20 in Heart Pool and Mantrap Spring, but it is also possible that ecotype demarcation could have incorrectly lumped sequence variants that belong to ecologically distinct populations. For instance, the PEB20 variants in question may be adapted to different pH, but are classified as members of the same PE by Ecotype Simulation 2. Microorganisms exhibiting patterns of island biogeography have also been observed in hot spring archaeal populations (Whitaker et al., 2003) and in a symbiotic fungus (Peay et al., 2010).

Most PEs were cosmopolitan with respect to basins (e.g., PEA66 and PEB93), and the most abundant variant of a PE was found in most springs within a basin, which could have been the result of frequent migration. Overlap of sequences in some PEs across the springs of a basin suggests that such ecotypes may disperse more readily than others, possibly because they inhabit the mat surface. Alternatively, such ecotypes may be most capable of occupying a niche in multiple, chemically similar springs. Populations might experience local extinctions due to the ephemerality of the hot spring (e.g., springs periodically dry up Brock, 1978; Fouke, 2011), interaction with a phage, or any other number of calamities that could plague a bacterium. The ephemerality of some hot springs, particularly those in the Mammoth basin, may provide an excellent resource for testing source-sink dynamics in bacterial communities.

The *Synechococcus* ecotypes of our study may disperse across springs by various mechanisms, including the aerosolization of microbes (Bonheyo et al., 2005), transport by one of the Yellowstone brine flies (*Ephydra* spp.) that live and feed on the mat (Brock et al., 1969), or perhaps by biologists studying hot spring inhabitants as the Genotype plus Boeing model suggests (Cohan and Perry, 2007). Rare dispersal events could be indicative of investigator-mediated contamination or natural events that happen rarely. The brine fly hypothesis is especially compelling because flies are known vectors for microbial transport (Markus, 1980; Junqueira et al., 2017), and different species of brine flies in YNP are known to distribute differently based on the pH and temperature of the spring (Resh and Barnby, 1984), ensuring that transported microbes are compatible with their new environment. Regardless of the mechanism, evidence is presented here that suggests a history of dispersal among springs within Yellowstone National Park, and infrequent recent and historical dispersal events between Yellowstone and Oregon hot springs.

The distribution of ecotypes predicted by Ecotype Simulation fits the expectations of insular biogeography. Springs that are more isolated have communities that are different from springs that are near each other. Our data suggests there are PEs that are endemic to a single basin and PEs that are more cosmopolitan. PEs endemic to a single basin may have specialized to the specific chemical environment provided by the water source of the springs in a basin, or may simply have been unable to migrate and become established elsewhere, diverging from parental populations over time. More cosmopolitan PEs may be generalists able to tolerate a variety of conditions, or may simply live in a position, such as the top of the mat, that may allow for easier migration between springs and subsequently basins.

## Data Availability Statement

The datasets generated for this study can be found in the NCBI Genbank, SAMN13631131, SAMN13631130, SAMN13631129, SAMN13631128, SAMN13631127, SAMN13631126, SAMN13631125, SAMN13631124, SAMN13631123, SAMN13631122, SAMN13631121, SAMN13631120, SAMN13631119, SAMN13631118, SAMN13631117, SAMN13631116, SAMN13631115, SAMN13631114, SAMN13631113, SAMN13631112, SAMN13631111, SAMN13631110, SAMN13631109, SAMN13631108, SAMN13631107, SAMN13631106, SAMN13631105, and SAMN13631104.

## Author Contributions

EB collected and analyzed barcode sequence data and assisted in preparation of the manuscript. JW analyzed barcode sequence data using Ecotype Simulation 2 and canonical correspondence analysis and aided in preparation of the manuscript. FC co-supervised the research and aided in preparation of the manuscript. DW obtained funding for the project, supervised the work, and participated in preparation of the manuscript.

## Dedication

This paper is dedicated to the memory of Richard W. Castenholz. Dick made the initial observations on cyanobacterial biogeography decades ago. His own contributions and his continuous encouragement, guidance, and assistance inspired and improved our work on this topic and on all aspects of the ecology of hot spring cyanobacteria. Dick's collegiality and friendly nature enhanced our enjoyment of doing science and set an excellent example for us all.

### Conflict of Interest

The authors declare that the research was conducted in the absence of any commercial or financial relationships that could be construed as a potential conflict of interest.

## Supplementary Material

Supplemental Figure 1 The number of shared sequences among all springs relative to the number of random sequences sampled in the total data set.
