# Supplementary material for: Biogeography of American Northwest Hot Spring A/B′-Lineage Synechococcus Populations
Source: Front Microbiol. 2020 Feb 24;11:77. doi: 10.3389/fmicb.2020.00077 (PMC7050468; doi:10.3389/fmicb.2020.00077)
Supplement: Supplemental Figure 1 — The number of shared sequences among all springs relative to the number of random sequences sampled in the total data set. [file Presentation_1.pdf]

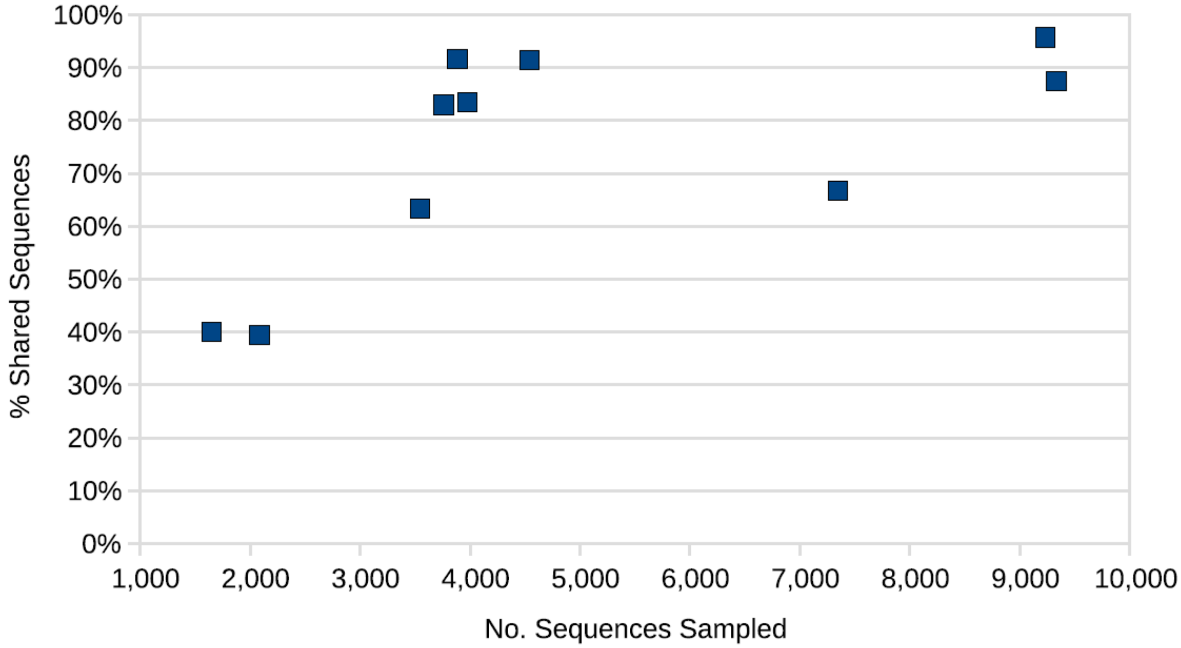

Supplemental Figure 1. The number of shared sequences among all springs relative to the number of random sequences sampled in the total data set.
